# Manufacturing Technology, Materials and Methods

**DOI:** 10.3390/ma19112218

**Published:** 2026-05-25

**Authors:** Rafał Gołębski, Antun Stoić

**Affiliations:** 1 Department Technology and Automation, Czestochowa University of Technology, Al. Armii Krajowej 21, 42-200 Częstochowa, Poland; 2 Mechanical Engineering Faculty, University of Slavonski Brod, Trg Ivane Brlić-Mažuranić 2, HR-35000 Slavonski Brod, Croatia

## 1. Introduction and Special Issue Scope

The topic of the Special Issue, “Manufacturing Technology, Materials and Methods,” was chosen in response to the growing importance of modern production technologies in shaping efficient, sustainable, and economically viable manufacturing processes. The dynamic development of material processing methods, manufacturing technologies, and engineering tools necessitates the integration of knowledge from materials science, production engineering, and process analysis. The motivation for addressing this topic is the need to identify and analyze phenomena accompanying production processes that influence the quality, durability, and efficiency of manufactured products. Of particular importance are issues related to machining, plastic forming, welding, surface engineering, and polymer and composite technologies, as well as research on tools, measurement systems, and inverse modeling. The Special Issue aimed to create a platform for the exchange of knowledge between the scientific and industrial communities and to present current research and technological solutions that respond to contemporary industrial challenges in terms of quality, reliability and optimization of production processes. The published papers cover the following topics in detail: the machining process, with particular emphasis on chip formation phenomena [1]; cold plasma processing and its impact on surface conditions after processing [2]; machining of hard materials in the context of assessing accelerated tool wear [3]; composite material coating technologies and assessment of wear resistance [4]; analyses of mold regeneration processes for improving their durability and wear resistance [5]; qualitative and technological aspects of modern gear manufacturing methods [6]; and the influence of lubricating and cooling fluids in slide-burnishing processes, as well as stereometric analysis of the surface after machining [7]. The material presented in the published articles is up-to-date and refers to current technologies and processes present in modern industrial plants.

## 2. An Overview of the Special Issue

In the first article, Joch et al. analyzed the effect of chip breaker geometry on the performance of monolithic rotating tools used in active rotary turning. Two tool variants were compared: standard and those equipped with an integrated chip breaker. The goal was to determine the effect of this modification on cutting performance, tool wear, cutting forces, and surface roughness of the workpiece. Cutting force measurements showed that the chip breaker tool generated higher force values in most cases. This was due to interrupted chip flow, increased friction, and local stress concentrations. At the same time, despite the higher mechanical loads, this tool exhibited lower wear. Reducing the contact time of the hot chip with the tool surface limited heat transfer, reducing thermal and abrasive wear. Surface quality analysis, however, revealed minimal impact of the chip breaker geometry on the Rz roughness parameter, maintaining comparable machining quality to the standard tool [1]. The use of a chip breaker improves control of the cutting process by effectively disintegrating chips and reducing tool wear. These benefits come at the cost of a slight increase in cutting forces. Appropriate selection of tool geometry and machining parameters allows for increased tool life and process stability without compromising the surface quality of the workpiece.

The second article by Bulakh presents research on the use of cold plasma as a modern method for reducing the roughness of the working surface of central plates in railway wagons. This technology represents a promising solution for surface treatment, enabling improvement in the quality of the surface layer without significantly affecting the mechanical and chemical properties of the material. The aim of the study was to evaluate the effectiveness of the process and determine its impact on the structure and performance parameters of alloy steel used in the railway industry [2]. The experiments involved measuring surface roughness before and after cold plasma treatment, and analyzing the microhardness, microstructure, and chemical composition of the samples. The obtained results confirmed the high effectiveness of the method in surface smoothing. The use of cold plasma effectively reduces the surface roughness of alloy steel without significantly affecting its microhardness or chemical composition. This method can significantly increase the durability of railway wagon components and reduce production and repair costs. The obtained results confirm the technology’s significant industrial potential, particularly where high surface smoothness is required while maintaining the material’s original properties.

Cutting processes involve many technological parameters that influence machining efficiency, with varying impacts on individual performance indicators. This complicates the selection of optimal tool operating conditions and the assessment of the importance of individual parameters while simultaneously considering multiple criteria. In the study presented in the third article, Zhao et al. [3] analyzed the milling process of a titanium alloy using an end mill. The aim of this study was to determine the effect of cutting parameters on tool wear and material removal rate, as well as to develop an effective method for comprehensively assessing these relationships. Experimental and analytical studies determined tool flank wear values and material removal rate indicators. Additionally, the nature of tool wear changes at subsequent machining stages was analyzed. A dynamic, comprehensive method based on a dual-stimulus model was used to assess the significance of technological machining parameters. It also took into account the variability of performance indicators over time and their varying importance. Cutting speed had the greatest impact, while depth of cut had the smallest [4]. Furthermore, a range analysis confirmed the consistency of the results with the comprehensive assessment, indicating the high reliability of the method used. The dynamic comprehensive assessment method is an effective tool for analyzing titanium alloy milling processes and selecting optimal cutting parameters. Cutting speed proved to be the most significant factor influencing process efficiency, while depth of cut was the least significant. The obtained results can support practical optimization of difficult-to-cut materials and improve tool life.

Growing environmental protection demands are driving the search for new, more sustainable production technologies. This is particularly important in the wood industry, which relies on wood as a limited natural resource. This growing demand requires maximum utilization of the raw material and reduction of material losses. In the fourth paper Olenska et al. analyzed the feasibility of using chip less wood peeling technology to produce base layers for flooring composites [5]. The composite base layers were made from pine veneers of various quality grades, including the lowest-grade material. Samples were then subjected to three-point bending tests to determine the modulus of elasticity and stiffness, which are key indicators of flooring material quality. The results demonstrated that the composite production process based on wood peeling is stable and produces repeatable results without the need for prior quality selection of the raw material. It was found that the base layer made of veneers obtained by rotary cutting exhibited better mechanical properties than the layer produced by sawing. The technology for producing composite flooring based on wood peeling is an effective and ecological solution [6]. It allows for the use of raw materials without quality selection, reduces waste, and lowers quality control costs. Simultaneously, it ensures high mechanical parameters of the finished products. The results confirm the significant industrial potential of this method as an alternative to traditional sawing-based production.

Regeneration of dies by hard facing worn surfaces is an effective and economically viable method for extending the service life of tools used in heavy industry [7]. This is particularly important in the production of wagon wheels, where dies operate under conditions of high mechanical and thermal loads, and intense wear. The aim of the presented research by Iovanas and Dumitrescu in the fifth paper is to comparatively evaluate the durability of dies made using the traditional method and dies reconditioned by hard facing the working layer using a specialized electrode. In the case of hard-faced dies, the deterioration process was more stable and uniform, demonstrating improved resistance to wear and tear. Advantages of hard-facing technology were also identified, such as low implementation costs, the possibility of automation, variable working-layer thickness, and the ability to fabricate bodies from less expensive materials. Limitations included unevenness of the hard-facing layer and the possibility of structural changes in the base material due to high temperatures, which may require additional heat treatment. Hard facing of working surfaces significantly increases the durability of dies used in the production of wagon wheels and reduces the costs associated with tool replacement [8]. This process allows worn components to be restored to full operational efficiency, supporting rational resource management and waste reduction. Properly designed remanufacturing is an effective tool for improving the reliability and efficiency of industrial processes.

Gears are fundamental components of drive systems and are widely used in the mechanical engineering, automotive, energy, aerospace, and precision machinery industries. Their importance is determined by high efficiency, high torque capacity, durability, and precision. However, gear production requires advanced production technologies and precise quality control. Thus, the authors of paper number six presented analyses of current trends in the design, machining, and measurement of gears, with particular emphasis on CNC machine tools, high-performance technologies, and additive methods. Changing the gear type or modifying the tooth profile often requires the purchase of new tooling. The development of multi-axis CNC machine tools has enabled most operations to be performed on a single workstation [9]. Thanks to HSC techniques, trochoidal strategies, and single-set machining, it is possible to turning, drilling, milling the gear, and finish after hardening without the need for multiple setups. This reduces costs, shortens preparation time, and increases manufacturing accuracy. Coordinate measuring machines equipped with specialized software dominate quality control, enabling the assessment of parameters in accordance with applicable tolerance standards. Gear production is evolving from conventional methods towards integrated CNC systems and digital technologies. Modern multi-tasking machine tools increase flexibility, shorten production times, and lower costs. At the same time, the development of additive manufacturing methods opens up opportunities for the effective regeneration of worn components [10]. Precise metrology remains crucial, ensuring product compliance with quality requirements.

Finally, the authors of the last paper examined the effect of the type of process fluid used during slide burnishing on the properties of the surface layer of C45 steel. This process is a finishing plastic forming method used to improve surface quality and extend the service life of machine components. The analysis assessed 2D and 3D roughness parameters, surface topography, the Abbott–Firestone curve, microhardness, and surface free energy (SFE). The obtained results confirmed significant improvements in the surface geometric parameters. The Ra value decreased by more than eight times, and the Rt value by more than five times compared to the post-grinding state. This indicates effective surface smoothing and reduced unevenness height. The Rpk, Rk, and Rvk parameters were also reduced. A reduction in Rpk and Rk indicates a flattening of the surface irregularities and improved load-bearing capacity of the surface layer [11]. At the same time, a 45–60% reduction in Rvk may indicate a reduced ability of the surface to retain lubricant, which should be considered in tribological applications. 3D topography analysis revealed deformation of the micro-irregularities and a more even Abbott–Firestone curve. Burnishing also increased the surface microhardness by a maximum of approximately 25%, with a hardened layer depth of up to 20 µm. Sliding burnishing significantly improves the surface layer of C45 steel by reducing roughness, increasing microhardness, and improving surface load-bearing capacity [12]. The choice of cutting fluid has a decisive impact on the final process outcome. The most favorable properties were achieved with an oil mixture with PMM and MoS_2_, recommended for components operating in friction pairs.

## 3. Conclusions

This Special Issue contains seven scientific papers presenting current information in the field of manufacturing technology, research on phenomena and factors influencing production processes, and new trends in engineering measurements. The content presented in the articles provides important information for engineers, researchers, and technologists in the field of modern manufacturing technologies and measurement techniques. The presented works demonstrate the growing prospects for the development of production technologies, linked to the ongoing automation and digitization of processes, the implementation of intelligent monitoring systems, and the search for materials and methods that increase energy efficiency and reduce the environmental impact of production. The Special Issue created a platform for presenting modern research and solutions that respond to the contemporary needs of industry and the scientific community. The included modern research and technological solutions indicate the dynamic development of manufacturing technologies and measurement techniques, which are increasingly responding to the requirements of modern industry in terms of automation, digitalization and sustainable development. The presented works emphasize the importance of intelligent process monitoring systems, advanced measurement methods, and innovative materials and production technologies that increase energy efficiency and reduce the negative impact of industrial activity on the environment. The research results can provide a basis for the further development of modern production systems, support the implementation of Industry 4.0 concepts, and inspire the creation of more precise, efficient, and ecological technological solutions. The Special Issue also confirms the important role of cooperation between the scientific and industrial communities in knowledge transfer and the implementation of innovative technologies in engineering practice.
